# Surface Monitoring of an MSW Landfill Based on Linear and Angular Measurements, TLS, and LIDAR UAV

**DOI:** 10.3390/s23041847

**Published:** 2023-02-07

**Authors:** Grzegorz Pasternak, Janina Zaczek-Peplinska, Klaudia Pasternak, Jacek Jóźwiak, Mariusz Pasik, Eugeniusz Koda, Magdalena Daria Vaverková

**Affiliations:** 1 Institute of Civil Engineering, Warsaw University of Life Sciences—SGGW, Nowoursynowska 159, 02-776 Warsaw, Poland; 2 Faculty of Geodesy and Cartography, Warsaw University of Technology, Pl. Politechniki 1, 00-661 Warsaw, Poland; 3 Department of Imagery Intelligence, Faculty of Civil Engineering and Geodesy, Military University of Technology (WAT), 00-908 Warsaw, Poland; 4 Department of Applied and Landscape Ecology, Faculty of AgriSciences, Zemědělská 1, Mendel University in Brno, 613 00 Brno, Czech Republic

**Keywords:** point cloud, landfill, unmanned aerial vehicle, terrestrial laser scanner, aerial laser scanning, geotechnical structure

## Abstract

Surface monitoring of landfills is crucial not only during their operation but also for later land restoration and development. Measurements concern environmental factors, such as leachate, migration of pollutants to water, biogas, and atmospheric emissions, and geotechnical factors, such as stability and subsidence. Landfill subsidence can be measured using modern surveying techniques. Modern measurement methods for landfill body displacement monitoring and their control after restoration and adaptation as recreational areas include terrestrial laser scanning (TLS), and scanning and low-altitude photogrammetric measurements from an unmanned aerial vehicle (UAV). The acquired measurement data in the form of 3D point clouds should be referenced to the local control network to enable a comprehensive analysis of data acquired using various techniques, including geotechnical sensors such as benchmarks, piezometers, and inclinometers. This study discusses the need for surface monitoring of municipal solid waste (MSW) landfills. A properly 3-D mapped landfill mass is the basis for ensuring the geotechnical safety of the restored landfill. Based on archival data and current measurements of the Radiowo landfill (Poland), this study compares the advantages and limitations of the following measurement techniques: linear and angular measurements, satellite measurements, TLS, and UAV scanning and photogrammetry, considering specific conditions of the location and vegetation of the landfill. Solutions for long-term monitoring were proposed, considering the cost and time resolution necessary for creating a differential model of landfill geometry changes.

## 1. Introduction

Mass movements and failures of municipal and post-production landfills, landslides in open-pit mines, or bulk product storage areas cause serious economic losses and safety hazards for workers during landfilling and for the public after landfill restoration [1,2,3]. Potential damage resulting from insufficient compaction of waste layers and inadequate slope protection requires continuous monitoring of the surface of municipal solid waste (MSW) landfills. Early detection of displacement of slowly creeping material is an important part of early warning against landslides. Stability assessment and prediction of large deformations and failures, including in open-pit mines and slopes during construction works, are usually performed using surveying techniques [4,5].

The requirement for geodetic monitoring for aforementioned facilities in Poland is set forth in the Regulation of the Minister of Environment of 30 April 2013, on landfills [6]. The regulation specifies the minimum frequency of geodetic measurements at a landfill every 3 months during the operational stage and every 12 months during the post-operational stage. The scope of monitoring should include control of the subsidence of the surface of the structure using geodetic methods based on measurements of displacements at control points, and evaluation of slope stability using geotechnical methods.

The stability of slopes can be achieved by using retaining walls, weight-bearing embankments, local slope mitigation, local replacement and recompaction of waste in the road substructure, and horizontal reinforcement with geogrid and car tire mattresses [7,8]. Accurate mapping of the surface of the restored landfill provides the starting material for the design of the target land use, such as forestry, agriculture, construction, parks, and recreation. In the case of the Radiowo landfill (Poland) selected for this study, the plan is to develop part of the area for sports and recreation and another part (slopes) for the installation of photovoltaic panels (PP) and wind turbines (RES). Given the large size of the landfill, steep slopes, and deformable material prone to landslides, it is necessary to measure the current shape and deformation of the mass over time to ensure geotechnical safety, preferably covering the entire landfill surface [9,10,11].

Landslide monitoring uses a number of surveying techniques and remote sensing tools to detect surface deformation at various scales and magnitudes. These include the Global Navigation Satellite System (GNSS), total station surveying [12], ground-based interferometric synthetic aperture radar (GB-InSAR), thermal infrared imagery [13], terrestrial laser scanning (TLS) [14], and techniques using UAVs [15,16,17,18].

The classic surveying techniques (GNSS, leveling, total station) allow obtaining results with high accuracy, but only at certain points, in a discrete manner, without depicting changes over the entire area. In contrast to these surveying techniques, laser scanning and photogrammetric techniques cover the entire monitored area with a dense grid of points, while being fast and efficient [19,20,21]. These techniques are widely used in various fields of geology/geomorphology, in issues related to landslide monitoring [22] and landslide risk assessment [16,17,23,24,25,26], for acquiring a high accuracy terrain model or making precise volumetric measurements.

Unlike traditional methods, monitoring using 3D laser scanning allows for non-contact point measurement, so it can complement traditional monitoring methods, improve the efficiency of slope monitoring, and provide data support/supplementation for site protection [4]. For example, Huang et al. [14] proposed a method for monitoring slow-moving landslides using long-range TLS. The proposed method features several key steps of TLS point cloud processing, including ground point extraction based on multidimensional classification, hybrid-weighted iterative closest point (HWICP) algorithm, and ALC2M-based deformation calculation [14]. Numerous literature examples indicate that TLS is an effective solution for providing accurate information on the movement of inaccessible areas characterized by landslide hazards [27,28,29,30]. In view of the expectations of planners and engineers designing both sports and recreational facilities, as well as those related to renewable energy sources, the use of appropriate/efficient measurement techniques provides the starting material for further work and enables the monitoring of phenomena occurring inside the landfill and on its surface.

The purpose of this paper is to analyze the possibility of using modern measurement techniques in monitoring the restored Radiowo landfill near Warsaw (Poland). The landfill was turned into a recreational and winter park with a slope for skiers and tobogganers, and in the summer for cyclists, runners, modelers, etc. The current geodetic monitoring of the site is based on point measurements of specially stabilized points (benchmarks) using GNSS methods and does not fully reflect the changes occurring in the entire area of the landfill, especially in the area of the ski run. The inventory measurement of this slope, a method that can serve as a baseline measurement for monitoring using point cloud analysis methods, is the focus of the article. Due to the specific conditions of the landfil’s location (proximity to the airport and protected areas), the existing vegetation, and the large size of the landfill (area of 17.3 hectares, height of more than 60 m), the paper proposes three alternative measurement methods. Two UAV-based survey methods, i.e., LIDAR and low-altitude photogrammetry, were first proposed. However, the close proximity to the airport imposes certain limitations and requirements for permits issued by Airport Flight Information Service (AFIS) may pose a serious problem in the context of long-term monitoring of the landfill. For this reason, TLS has been proposed as an alternative. The paper compares the measurement results of these methods in the form of point clouds with reference data obtained from linear and angular measurements (trigonometric leveling).

## 2. Study Area

**Figure 1:**
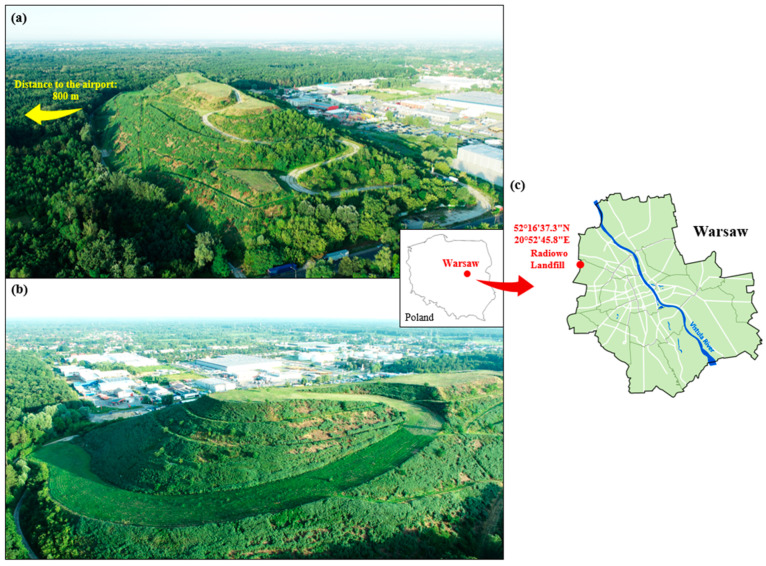
Location of the Radiowo landfill in Warsaw: (**a**) view from the access road (northeast slope), (**b**) view from the ski run (southeast slope), and (**c**) location of the landfill within the administrative borders of Warsaw.

The study concerned the Radiowo landfill site in Warsaw in July 2022. The site is located near the northwestern border of Warsaw at the junction of the Bemowo district and the Stare Babice municipality (Figure 1). The area of the landfill is about 17.3 hectares, and its height relative to ground level is about 60 m [31].

Initially, the Radiowo landfill received only unsegregated MSW (1961–1991). Due to the unfavorable location in terms of geotechnical conditions (wetlands) and the occurrence of numerous slope landslides, from 1992 the landfill began to accept only solid waste generated from compost produced at the Radiowo composting plant (foils, tires, textiles, scrap metal), and, from 2012 to 2018, stabilized compost produced by the mechanical-biological processing of MSW. Since 1993, to reduce the slopes and thus improve their stability, a number of reclamation works have been carried out, which were completed in 2018. Since then, cyclic geodetic monitoring on installed benchmarks has made it possible to assess the condition of the structure’s geometry in terms of horizontal displacements and subsidence. This process was aimed at preparing the landfill, which was closed in 2017, for its transformation into a public facility, i.e., a ski run for skiers, snowboarders, and tobogganers, as well as runners and cyclists [7,32]. The regulation [6] allows building constructions on the crown of a landfill after fifty years from the date of its closure. However, it allows a shorter time based on geotechnical and sanitary expert opinions in cases where this does not endanger human life and health or cause a risk to the environment.

The lump of the Radiowo landfill was monitored for many years by measuring controlled points using traditional surveying methods (more than 100 benchmarks were installed), which over time were supplemented by satellite measurements using GNSS receivers. The survey work at the Radiowo landfill used a survey set consisting of two GNSS Real Time Kinematic (RTK) receivers operating in a Base and Rover mode [8]. In such a configuration, one of the receivers serves as a stationary base and sends corrections via radio to the other mobile receiver (Rover) performing the measurement. The advantage of such a solution is that a single base station can cooperate with several mobile receivers which serve as the equipment for controlling construction machines, such as dozers, excavators, and graders used in earthmoving works at the landfill [7].

## 3. Materials and Methods

### 3.1. Periodic Archival Measurements

In the past, the studied landfill experienced frequent landslides caused by improper compaction of waste layers and inadequate protection of steep slopes. Accordingly, landslide areas have been rebuilt in accordance with earthwork rules and considering slope stability conditions (horizontal reinforcements, retaining structures), and geodetic monitoring has been introduced. Periodic geodetic monitoring of control points allows early detection of potential hazards and early response to the occurring changes in the shape of the landfill, especially in the areas of slopes with landslide hazards.

**Figure 2:**
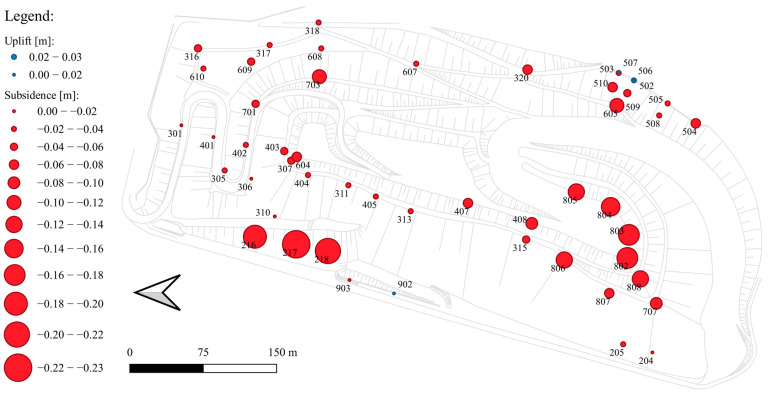
Map of annual subsidence and uplift of control points (December 2020–December 2021) [7].

During the 30 years of geodetic monitoring, a total of 105 control points (benchmarks) were established, 59 of which were monitored for more than 5 years. Some of them have existed since the beginning of the site’s monitoring, i.e., since 1993, and are located on the northern and western slopes of the landfill. Long-term monitoring has allowed the assessment of the mechanical parameters of the waste (deformation and strength) and the prediction of the displacements occurring at the landfill [8]. Figure 2 shows the location of the control points and the values of their measured subsidence over a one-year period: December 2020–December 2021.

As of 2017, i.e., after the formation of the target landfill body, the largest overall subsidence has been about 1.8 m and applies to the southern slope formed along the designed ski run. This area is still subject to increased subsidence, which is visualized in Figure 2 in the form of circles with a diameter corresponding to local displacement. The value of displacements per year reaches about 0.20 m [7]. The subsidence is expected to slowly disappear over time; however, it may locally increase following the installation of facilities on the surface of the landfill (buildings, PP, pole structures, etc.).

### 3.2. Data Sets

In the area of the landfill in 2022, the periodic measurements were made using four methods: (i) Method 1 (set 1)—photogrammetric measurements from an unmanned aerial vehicle (UAV); (ii) Method 2 (set 2)—aerial laser scanning (ALS); (iii) Method 3 (set 3)—terrestrial laser scanning (TLS); and (iv) Method 4 (set 4)—linear and angular measurements.

**Figure 3:**
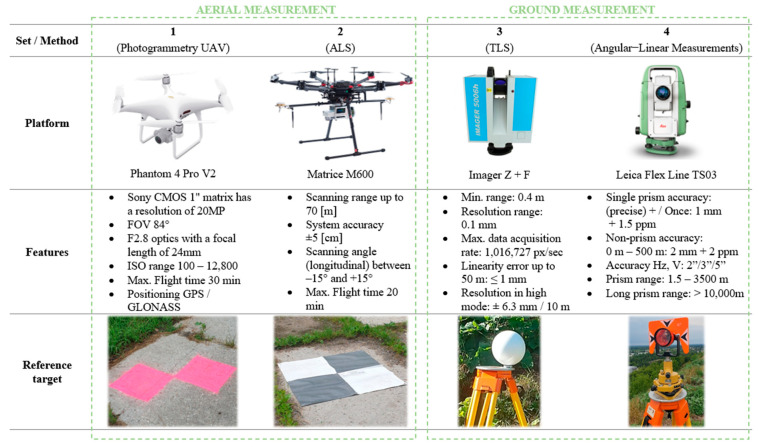
Overview of the research measurements. Developed based on [33,34,35].

As a result of the measurement using methods 1–3, point clouds were obtained, differing in density and resolution from the recorded data. Figure 3 summarizes the general technical and accuracy characteristics of the instruments and reference points used.

Set 1: Digital images were obtained from a photogrammetric flight taken using a Phantom 4 Pro platform, which is equipped with a 1-inch, 20-megapixel CMOS sensor with a 24-mm FOV lens. The measurements were taken from a height of 60 m above the landfill crown. The BSP measurement was divided into five flights. During the first flight, vertical (nadir) images were taken. In the next four, flights were carried out with oblique photos in four different directions (north, south, east, and west). A total of 18 longitudinal and 18 transverse series were taken. Coverage of longitudinal and transverse images was 80% for nadir images and 50% for oblique images with an off-nadir angle of 30°. A total of 1768 images were acquired. In the field, before the flights, 13 ground control points (GCPs) were marked and their XYZ coordinates were measured. Their shape, color, and size enabled unambiguous identification of the points in the field. The point clouds from imaging data were generated using the Pix4D Mapper software.

Set 2: Data acquired with an airborne laser scanning system using a DJI Matrice M600 platform. The flight was performed at an altitude of 60 m above the crown of the landfill. The distance between each flight path was 20 m. A total of four overflights were performed. The S50 LiDAR system mounted on the platform consists of a scanner: Velodyne’s VLP-16 and a Sony A6000 RGB camera. The survey can be performed at a distance range of up to 70 m with an accuracy of ±0.05 m and a scanning angle of −15° to 15° (longitudinal). In the field, 10 control points were marked in the form of dedicated reference mats prior to the flights (Figure 3). During the flight, a GNSS reference station was used to reference the data. The average density of the resulting point cloud was about 160 pts/m^2^. In areas of double coverage, the point cloud density was 200–250 pts/m^2^.

Set 3: Point clouds (9 scans) acquired with the Z + F Imager 5006H laser scanner. The clouds were recorded in XYZI format (where I denotes the intensity of reflection of the laser beam from the measured structure). Benchmarks were the spheres with known diameters situated on geodetic tripods. The measurement was performed with high resolution reaching ±6.3 mm/10 m. The data acquisition process included several steps: (i) planning the measurement stations; (ii) arrangements of reference spheres for surveying tripods; and (iii) landfill scanning. The oriented point cloud was developed (filtering, referring the point cloud into a global coordinate system) in Faro Scene software.

Set 4: Reference data: In order to reference measurements made by alternative methods (set 1, set 2, and set 3), the position of control points located on the landfill was determined using trigonometric leveling. The measurement was made using the Leica Flex Line TS03 total station. To ensure the highest possible accuracy of the measurement, the so-called “three tripod method” was used. The measurement was referred to reference points located outside the area of the structure’s influence.

### 3.3. Data Processing

#### 3.3.1. Method 1

The processing of the images acquired from the photogrammetric flights was performed in Pix 4D Mapper software. It consists of calculating the internal and external orientation elements of each photo by generating tie points. This stage as well as the process of generating a dense point cloud is based on multi-image correlation (Dense Matching).

The alignment of the photo block was carried out based on the signaled matrix measured in the field—the ground control points (GCPs). After measuring the photopoints, a dense point cloud was generated. A GSD value of 0.03 m was obtained. The average density of the point cloud was 185 points per 1 m^2^.

**Figure 4:**
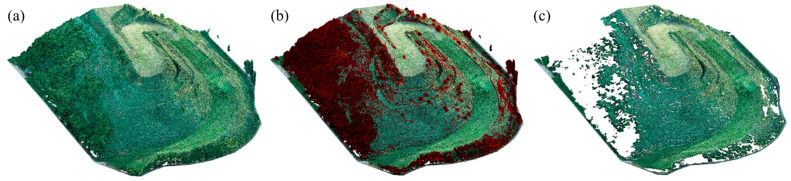
Point cloud generated from photos showing (**a**) green vegetation visible on the landfill, (**b**) the process of vegetation filtration, and (**c**) the surface of the site after filtration.

An important step in the processing of point clouds was their filtering, resulting from the intensive coverage of slopes with vegetation. Figure 4 shows an example of vegetation filtering of photogrammetric data. The landfill area is largely covered with vegetation, especially in the western part, as indicated by the red color in Figure 4b.

#### 3.3.2. Method 2

The processing of ALS data was performed using LIDAR 360 software, which consisted of georeferencing the point cloud based on the position of the GNSS ground antenna and applying appropriate data filtering. Points that were recorded during the UAV’s turnaround, ascent, and descent were removed from the dataset. Redundant points covering data areas recorded in overflights on adjacent paths were eliminated. Points recorded by beams emitted at angles greater than 25° from the nadir were removed. Vegetation filtering was also performed, as well as correction of the mutual position of fragments of the point cloud registered during the flight on two adjacent parallel paths (elimination of the so-called “broadside error”).

#### 3.3.3. Method 3

The acquired scans were recorded in local coordinate systems. The first step in processing the TLS data was to orient the scans. This process was performed based on reference spheres and points homologously located on adjacent scans using Faro Scene software. The accuracy of the scan orientation results is affected by (i) the incidence angle of the laser beam; (ii) surface color; (iii) the type of surface material; and (iv) the number and geometry of overlapping points on several scans.

Based on the measured GCPs, the resulting point cloud was transformed into the coordinate system in which the other measurements were made (EPSG:2178) and was filtered to remove vegetation from the image.

#### 3.3.4. Method 4 (Reference Method)

The results of the angle-line measurements were used to calculate the elevation and topographic position of the control points. Calculations of the angular–linear network were made using the WinKalk program using the least squares method. Separate calculations were made for the altimetric network and horizontal geodetic network for topographic measurements. The average error in the calculation of the height of the points was 4 mm, while the average error in the topographic position was 25 mm.

## 4. Methodology

The study used four data sets (sets 1–4), acquired using different measurement systems. The measurements focused on the areas of the access road to the crown of the landfill (northern part), the crown of the landfill, and the ski run located in the southeastern part of the landfill. The alternative measurement methods proposed in the paper (sets 1 and 2) were compared with classical methods (set 4). Due to the large area of the landfill, TLS measurements were used only to image the western, less vegetated part of the landfill which is, however, the main area exposed to the landslide associated with the use of the slope for recreation in winter (ski run). The comparison of measurement methods was performed in the vertical plane, keeping in mind the results of archival measurements and the trends of change determined based on the analysis of behavioral models of similar objects, indicating changes in elevation as the main components of the displacement of control points on the site.

The study proposed three approaches to compare the methods used: (i) Approach 1 based on comparing the altitude of homologous points; (ii) Approach 2 based on the direct comparison of point clouds using the cloud-to-cloud (C2C) method; and (iii) Approach 3 based on the comparison of cross-sections created from point clouds.

**Figure 5:**
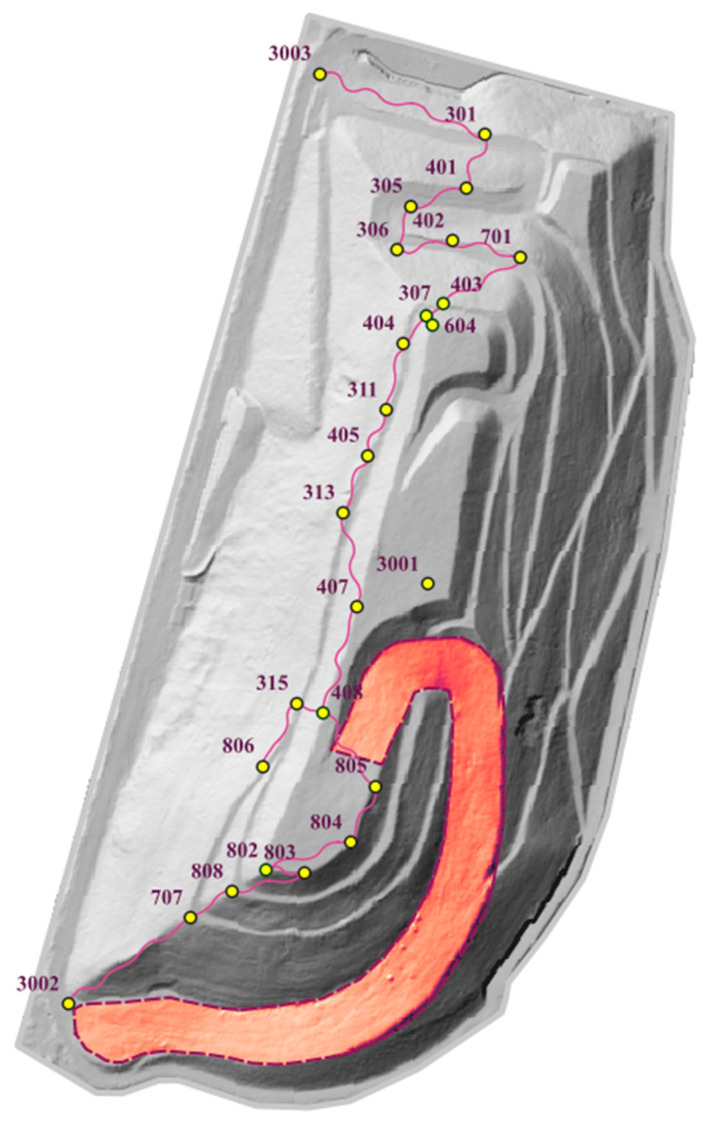
Location of the analyzed homologous points and the ski run.

Approach 1: To carry out a comprehensive analysis of the point clouds in the landfill area, the height differences determined at the control points based on the heights measured on the point clouds (obtained from methods 1 and 2) and the heights measured by method 4 (the reference data) were compared. The analyzed points are located at different locations in the landfill area (Figure 5). These include points located on the concrete road (the northern part of the landfill), among the vegetation along the road, on the slopes (mainly on the western side of the site) and on the crown of the landfill. Figure 5 shows the location of the control points measured using method 4.

Approach 2: TLS measurement was limited to the ski run located in the southeastern part of the landfill. The range of measurements is marked in red in Figure 5 (bounded by a purple dashed line). Only cloud-to-cloud distance analyses obtained from methods 1–3 were performed in this area.

Approach 3: When studying the quality of point clouds, attention should also be paid to their detail level related to their resolution [36]. The assessment of their detail levels was based on a comparative analysis of the profiles created from the measurements. In this approach, cross-sections along the ski run were extracted and their geometry was compared for each of the three-point clouds (sets 1–3).

## 5. Results and Discussion

Approach 1: This approach presented a comparison of point clouds obtained by methods 1 and 2 with reference data (set 4). The analyses performed included a comparison of height differences on the control points and a comparison of the difference in height between adjacent points, which are marked with a purple line in Figure 5. The results are shown in Table 1 and Table 2.

The largest height differences (0.20–0.27 m) were obtained for points 3003, 301, 401, and 604 located in the northern part of the landfill on the road or in its close location to the slopes amidst dense vegetation. In other areas of the landfill, the differences were smaller. In general, the recorded height differences did not exceed 0.28 m. The standard deviation was 0.12 m. The results obtained for the area excluding the northern slope were sufficient and in accordance with the requirements for the accuracy of topographic-elevation measurements for earth structures.

An analysis of elevation differences was also conducted for selected sections located between control points, and the results are shown in Table 2.

Maximum height differences were obtained for sections 307–404 and 311–405. These sections are located in the northern part of the landfill crown in an area covered with vegetation. The value of the standard deviation was 0.12 m. The obtained values of height differences did not exceed 0.30 m.

**Figure 6:**
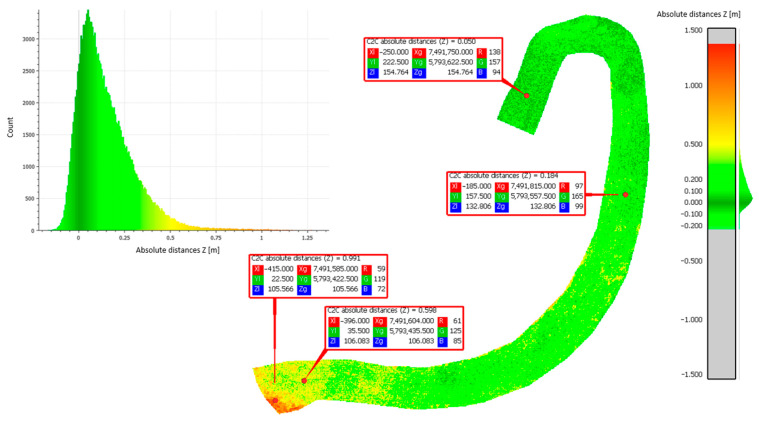
C2C distance analyses for set 1 compared to set 2.

**Figure 7:**
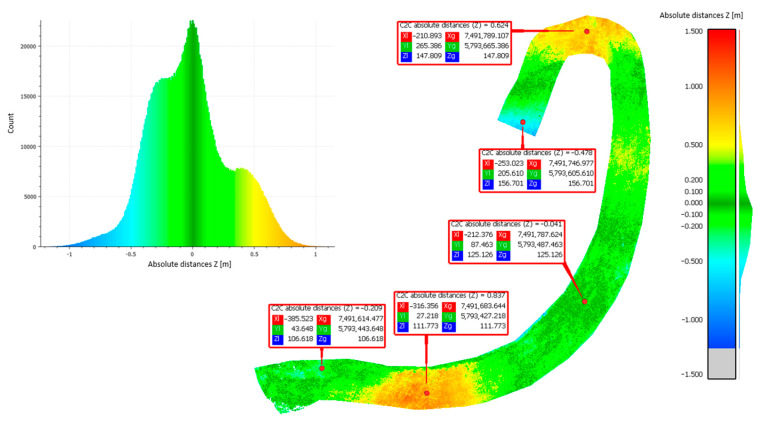
C2C distance analyses for set 2 compared to set 3.

**Figure 8:**
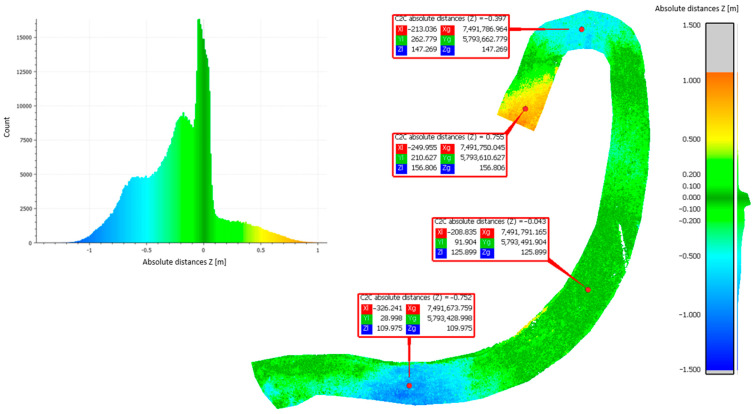
C2C distance analyses for set 3 compared to set 1.

Approach 2: Cloud-to-cloud distance analyses were performed to calculate the average distance between point clouds derived from methods 1–3. This approach adopted the principle of comparing the results side by side. The differences in the distances of the resulting point clouds are shown in Figure 6, Figure 7 and Figure 8.

Table 3 shows the statistical data calculated using the Gaussian elimination for the mean value of the height differences obtained from the comparison of the different methods, as well as their standard deviation (mean value, standard deviation).

Based on the obtained results of measurements and approximations, the mean value of the height difference for the compared methods was 0.20 m (set 1 vs. set 2), −0.02 m (set 2 vs. set 3), and −0.21 m (sets 3 vs. set 1), respectively. The largest average differences were observed in the comparison of set 1 (photogrammetric method). Methods 2 (ALS) and 3 (TLS) are based on laser scanning to penetrate vegetation and better reach the ground surface, while method 1 is based on surface measurement. Therefore, the difference may be due to the vegetation cover lushly growing on the landfill during the summer. The standard deviation values were 0.15 m (set 1 vs. set 2), 0.32 m (set 2 vs. set 3), and 0.33 m (set 3 vs. set 1), respectively. The largest standard deviations were found when comparing set 3 acquired by using method 3 (TLS). This parameter indicates how widely the values of a given size are scattered around its mean. Its larger value for set 3 may indicate errors made in the mutual orientation of the measurement stations, as shown in Figure 7 and Figure 8.

**Figure 9:**
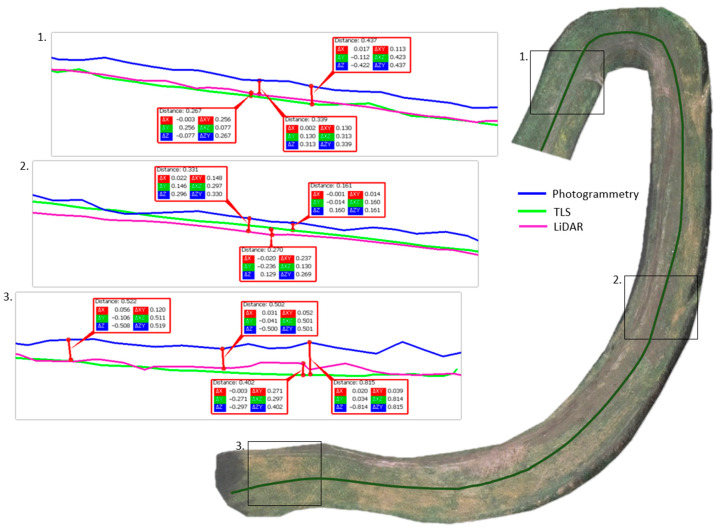
Height differences on selected profiles between point clouds extracted based on methods 1–3.

Approach 3: Point cloud analysis was carried out based on profiles generated in the landfill area. Figure 9 shows three selected profiles generated in the northern, central, and southern areas of the ski run axis. The profiles were generated from the acquired point clouds (sets 1–3). Strips of 0.5 m width were used in profile extraction.

Analyzing the generated land surface profiles it can be concluded that the greatest differences in elevation occurred between the data acquired using method 1 and method 2. This is due to the greater penetration of vegetation in method 2 and the surface measurement in method 1.

The highest accuracy of measurements at the Radiowo landfill was obtained in discrete linear and angular measurements (trigonometric leveling) of controlled points, which is due to the accuracy of the method itself, the precise measuring equipment used and the high regime of measurement execution (adequate repeatability of measurements of leveling sections—both in forward and backward leveling).

The quasi-continuous methods of measurement that produce point clouds can be an excellent complement to classical measurements. However, due to the accuracies of the methods themselves and the data filtering necessary due to vegetation, methods 1, 2, and 3 were consistent with each other in terms of accuracy (height differences of 0.20 m) but inferior compared to the GNSS RTK, classical linear and angular methods.

On the other hand, the main advantage of methods 1–3 over method 4 is the representation of the whole body of the geotechnical structure and not just the coordinates of selected individual points. Data in the form of a point cloud can provide a basis for modeling the shape of the solid of the entire landfill, not just periodic measurements of coordinates of control points.

However, it is necessary to compare not only the area coverage or economic context, but, above all, the accuracy and resolution of the acquired data and their further use in the post-restoration management of the landfill. In particular, the selection of the method of periodic and inventory measurement must consider the expected values of change and the purpose of the measurement. In particularly fortified areas that are the basis of the stability of the landfill (steep slope, access road, crown), periodic control measurements of displacements should be carried out discreetly in the network of control points using linear and angular methods, precision leveling or GNSS RTK or STATIC methods, which will allow achieving repeatability and accuracy in the range of single millimeters and centimeters (depending on the method). ALS and TLS methods are suitable in areas intended for intensive use and annual renewal of geometry (artificial ski run) to control displacements in decimeter ranges.

**Figure 10:**
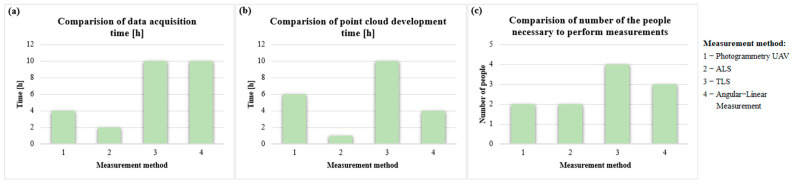
Comparison of the applied methods in terms of (**a**) data acquisition time, (**b**) point cloud development time, and (**c**) the number of people necessary to perform the measurement.

The use of selected measurement methods to inventory the landfill was compared from an economic point of view, considering the selected two aspects, i.e., time (to perform field work, conduct an intimate study) and the number of people required to perform the measurement, the results of the comparison of the proposed measurement methods are shown in Figure 10.

The analysis of the aforementioned data (Figure 10) shows a clear advantage of methods 2 and 1, both in terms of the time taken to perform the measurement, the indoor analysis, and the number of people required to perform the field measurements. The most accurate results were obtained using the trigonometric leveling method, despite only obtaining the coordinates of single points.

The photogrammetric method [16,17,18], the ALS method [15], and the TLS method [4,5,13,14,19,22,23] are successfully used in the periodic geodetic monitoring of facilities similar in their construction to MSW landfills (large-scale, soil-built facilities). These include open-pit mines, natural landslide slopes, post-production landfills, and various types of bulk product storage areas. On this basis, it can be concluded that these methods will also find application in the periodic monitoring of MSW landfills. The paper attempts to validate the above-mentioned methods in the area of the reclaimed MSW landfill Radiowo, which is a modern approach to a paper. The research is a prelude to a better understanding of modern measurement methods in the context of periodic monitoring of such facilities.

## 6. Conclusions

The analyses and comparisons of the measurement methods to control and inventory periodic changes at landfills described in the paper allowed identifying of their advantages and limitations in the context of long-term geodetic monitoring. In structures such as landfills, the expected and recorded annual displacements reach decimeters in the course of a year, and in multi-year cycles even several meters, depending on the way the slopes are reinforced and how they are used. The use of proposed remote sensing methods is justified mainly because of the magnitude of predicted displacements of the earth. The recorded height differences between the data set from the photogrammetric method and the data set from ALS did not exceed 0.28 m and the standard deviation was 0.12 m. The subsidence of the Radiowo landfill surface is variable. Its values depend on the morphology and age of the waste. In the last five years, the values of subsidence on the crown of the landfill have ranged from 0.6 to 2.1 m, giving an average annual increase range from 0.12 to 0.40 m. In the next ten years, average annual increments in subsidence are forecast to be between 0.02 and 0.10 m.

TLS-based monitoring allows for high-resolution data but is labor-intensive and not very economical in the context of monitoring the entire landfill. This method can be used to monitor selected portions of the landfill that are particularly vulnerable to displacement and in areas where high accuracy is required, for example, in the ski run or slopes with exceeded critical conditions. When performing such measurements, it is important to keep in mind the close location of measurement stations and as many benchmarks as possible, due to the vast area of the landfill and the lack of terrain details that hinder the process of orienting the scans.

Monitoring using UAVs (photogrammetry and ALS) can be an excellent complementation of the classical methods in monitoring periodic changes on a landfill, especially in areas where it is not possible to stabilize controlled points, or due to dense vegetation (used as slope reinforcement) that prevents good visibility between points. On the other hand, it cannot completely replace classical measurement methods based on cyclic measurement of control points, due to the required accuracy of displacement determination and current legal regulations in Poland (requirement to install and periodic measurement benchmarks).

Due to the growing seasons in which the quality of data and the need for filtration change significantly, and also due to changing atmospheric conditions (humidity: precipitation and haze), geodetic monitoring of landfills should be carried out in late autumn (late October/early November) or early spring (late March/early April). Comparative analysis of the acquired point cloud data shows the greatest differences in areas with high and dense vegetation. This difference can be seen especially in the results of the photogrammetric method compared to the ALS and TLS methods. The mean value of the height difference for the compared methods was 0.20 m (photogrammetric method vs. ALS), −0.02 m (ALS vs. TLS), and −0.21 m (TLS vs. photogrammetric method). High and dense vegetation makes it impossible in the photogrammetric method to directly measure the area of ground cover of the landfill.

The presented results of the 3-D measurements will be used during the design of facilities at the restored landfill, including sports and recreational equipment and renewable energy sources (PP, RES, and a landfill gas collection system). The obtained imaging will also be useful in the periodic assessment of the geotechnical safety of the structure and in designing appropriate strengthening solutions on the slopes of the landfill. Finally, they may be used as references for future inventory studies and visualizations of changes.

## Figures and Tables

**Table 1:** Summary of height differences at the control points.

Point No.	Height Differences (m)		
	∆H_4–2_	∆H_4–1_	∆H_4–2_–∆H_4–1_
3003	0.143	−0.067	0.210
301	1.225	0.996	0.229
401	0.507	0.295	0.212
305	0.699	0.648	0.051
306	2.009	1.908	0.101
402	0.858	0.778	0.080
701	0.326	0.173	0.153
604	−0.292	−0.018	−0.274
403	−0.019	−0.041	0.022
307	−0.665	−0.502	−0.163
404	0.031	−0.102	0.133
311	−0.184	−0.140	−0.044
405	−6.226	−6.392	0.166
313	−0.131	−0.188	0.057
407	−0.325	−0.341	0.016
408	−0.259	−0.103	−0.156
315	−1.135	−1.068	−0.067
806	0.098	0.109	−0.011
802	0.004	−0.067	0.071
803	−0.022	0.036	−0.058
804	0.241	0.231	0.010
805	−0.142	−0.043	−0.099
3001	0.033	−0.037	0.070
808	−0.875	−0.783	−0.092
707	−0.278	−0.280	0.002
3002	−0.212	−0.198	−0.014

**Table 2:** Summary of elevation differences on selected sections between control points.

Number of Section	Height Differences (m)					
	∆H_4_	∆H_2_	∆H_1_	∆H_4–2_	∆H_4–1_	∆H_4–2_–∆H_4–1_
3003–301	−11.866	−10.784	−10.803	−1.082	−1.063	−0.019
301–401	−8.460	−9.179	−9.161	0.719	0.701	0.018
401–305	−2.823	−2.631	−2.470	−0.192	−0.353	0.161
305–306	−4.893	−3.583	−3.633	−1.310	−1.260	−0.050
306–402	−2.072	−3.223	−3.202	1.151	1.130	0.021
402–701	−3.620	−4.152	−4.225	0.532	0.605	−0.073
701–403	−14.560	−14.904	−14.774	0.344	0.214	0.130
403–307	−2.290	−2.937	−2.751	0.647	0.461	0.186
307–404	2.113	2.809	2.513	−0.696	−0.400	−0.296
404–311	−0.285	−0.501	−0.323	0.216	0.038	0.178
311–405	0.882	−5.160	−5.370	6.042	6.252	−0.210
405–313	0.267	6.362	6.471	−6.095	−6.204	0.109
313–407	−8.591	−8.785	−8.744	0.194	0.153	0.041
407–3001	−0.668	−0.311	−0.364	−0.357	−0.304	−0.053
407–408	0.758	0.824	0.996	−0.066	−0.238	0.172
408–315	9.722	8.846	8.757	0.876	0.965	−0.089
315–806	0.259	1.492	1.436	−1.233	−1.177	−0.056
408–805	0.119	0.236	0.179	−0.117	−0.060	−0.057
805–804	−1.088	−0.705	−0.814	−0.383	−0.274	−0.109
804–802	0.500	0.263	0.202	0.237	0.298	−0.061
802–803	0.087	0.061	0.190	0.026	−0.103	0.129
803–808	10.314	9.461	9.495	0.853	0.819	0.034
808–707	9.965	10.563	10.468	−0.598	−0.503	−0.095
707–3002	35.242	35.307	35.324	−0.065	−0.082	0.017

**Table 3:** Results of statistical analysis using the Gaussian elimination.

	Sets 1 vs. 2	Sets 2 vs. 3	Sets 3 vs. 1
Mean (m)	0.201	−0.024	−0.209
Standard deviation (m)	0.153	0.321	0.329

## Data Availability

The data presented in this study are available on request from the co-author Grzegorz Pasternak, e-mail: grzegorz_pasternak@sggw.edu.pl.
